# An Improved Multi-Source Data Fusion Method Based on the Belief Entropy and Divergence Measure

**DOI:** 10.3390/e21060611

**Published:** 2019-06-20

**Authors:** Zhe Wang, Fuyuan Xiao

**Affiliations:** School of Computer and Information Science, Southwest University, No.2 Tiansheng Road, BeiBei District, Chongqing 400715, China

**Keywords:** Dempster–Shafer evidence theory, belief entropy, belief Janson–Shannon divergence, multi-source data fusion

## Abstract

Dempster–Shafer (DS) evidence theory is widely applied in multi-source data fusion technology. However, classical DS combination rule fails to deal with the situation when evidence is highly in conflict. To address this problem, a novel multi-source data fusion method is proposed in this paper. The main steps of the proposed method are presented as follows. Firstly, the credibility weight of each piece of evidence is obtained after transforming the belief Jenson–Shannon divergence into belief similarities. Next, the belief entropy of each piece of evidence is calculated and the information volume weights of evidence are generated. Then, both credibility weights and information volume weights of evidence are unified to generate the final weight of each piece of evidence before the weighted average evidence is calculated. Then, the classical DS combination rule is used multiple times on the modified evidence to generate the fusing results. A numerical example compares the fusing result of the proposed method with that of other existing combination rules. Further, a practical application of fault diagnosis is presented to illustrate the plausibility and efficiency of the proposed method. The experimental result shows that the targeted type of fault is recognized most accurately by the proposed method in comparing with other combination rules.

## 1. Introduction

Multi-source data fusion is the process of combining data obtained from different sources to make robust and complete evaluation on the certain system. As is known, single data source cannot provide sufficient information to detect a complex system in a full scale. By contrast, multi-source data fusion presents comprehensive and credible results after integrating groups of data that reflect various features of the system [1,2]. Therefore, multi-source data fusion technology is widely applied in many real applications, such as energy management strategy [3,4], health prognosis [5,6,7], suppliers selection [8,9,10,11], decision making [12,13,14], evaluation [15,16,17], etc. However, since the single data source is easily disturbed by environmental factors, it is unavoidable to meet the situation when the data collected from different sources are inconsistent, irrelevant or even conflicted [18]. How to fuse these groups of data from different sources correctly has received much attention but is still an open problem [19,20]. Thus far, many theories and methods have been proposed to solve the uncertain problem, which were extended from Z-numbers [21,22], D-numbers [23,24,25,26,27], R-numbers [28], fuzzy sets [29,30,31,32], rough sets [33], evidence theory [34], entropy [35,36], quantum [37], and so on.

Dempster–Shafer evidence theory (DS evidence theory), firstly proposed by Dempster [38] and later developed by Shafer [39], is a general framework for reasoning with uncertainty. As a generalization of Bayesian theory, DS evidence theory can express uncertain and imprecise data more explicitly by using mass function, which can assign the probability to the union of single elements [40,41]. Besides, DS evidence theory provides a combination rule to fuse pieces of evidence. Due to its flexibility and effectiveness on handling uncertainty, DS evidence theory is widely applied in information fusion technology [42,43,44]. However, the combination rule in DS evidence theory presents counter-intuitive results when evidence is highly in conflict [45,46]. To address this problem, many modified combination methods have been proposed, which derive from two basic strategies. One is to modify classical DS combination formula. Based on this strategy, Yager [47] believed that little about conflicting evidence can be understood and reassigned the conflict constant to the unknown space. In [48], Smets thought that the conflict is attributed to the incompleteness of the frame of discernment and proposed unnormalized combination rule. In [49], Lefevre et al. proposed a general framework to unify the general combination rules. In [50], Sun et al. believed that the availability of conflicting evidence is associated with their credibilities. In [51], Li et al. reallocated the conflict constant based on the weighted average support degree to each piece of evidence. However, the main shortcoming of these methods is the loss of associative properties, which greatly increases the computational complex degree especially when fusing thousands of pieces of evidence simultaneously. Another strategy is to pre-process the original evidence and apply the classical DS evidence theory on the adjusted evidence multiple times. Many combination methods have been proposed on the basis of this strategy. In [52], Murphy generated the modified evidence by simply averaging the original evidence. In [53], Deng et al. took the distance between pieces of evidence into consideration and reallocated the weight on the evidence. In [54], Jiang et al. proposed a new combination rule based on information volume calculated by belief entropy. In [55], Zhang et al. applied cosine theorem to calculate the support degree of evidence. In [56], Lin et al. generated a similarity vector by measuring Euclidean distances between pieces of evidence before generating the weighted average evidence. Although these combination methods presented quite reasonable fusing results, there is still some room for further improvement. In this paper, therefore, a novel multi-source data combination method is proposed to handle the problem of highly-conflicted evidence fusion.

In particular, by taking advantage of the belief entropy to quantify the information volume of the system and belief divergence to measure the difference among multi-source data, the credibility and the information volume, as two important factors of evidence are integrated to allocate the weight on the original evidence. In this way, the weight of untrustworthy evidence is declined, so that the influence of conflicted evidence is controlled more strictly. The main steps of the proposed procedure are concluded as follows. Firstly, the credibility of evidence is calculated according to their similarity with the average evidence. Besides, the belief entropy is applied to calculate the information volume of each piece of evidence. After that, the weight is allocated on the evidence based on their credibility and information volume before the weighted average evidence is generated. Finally, classical DS evidence is used to fuse the modified evidence multiple times and the final result is obtained. A practical application of fault diagnosis is given to prove the efficiency of the proposed method.

The following parts of this paper are organized as follows. In Section 2, some basic concepts and definitions of DS evidence theory, belief entropy and Belief Jenson–Shannon divergence are concisely introduced. In Section 3, a novel multi-source data fusion method is presented. In Section 4, a numerical example is used to compare the fusing results with other existing combination rules. In Section 5, the proposed combination method is applied in a practical example of fault diagnosis. Finally, the conclusion of this paper is discussed in Section 6.

## 2. Preliminaries

In this section, several preliminary theories are briefly introduced, including DS evidence theory, belief entropy and Belief Jenson–Shannon divergence.

### 2.1. Dempster–Shafer Evidence Theory

Dempster–Shafer evidence theory is an extension of the Bayes probability theory. Comparing with probability theory, DS evidence theory can not only assign the probability on one single element, but also on the subset of the universal set [57,58]. Besides, DS evidence theory can handle uncertainty and imprecision without prior probability is given [59,60,61,62]. When the probability is only allocated on several single elements, DS evidence theory degenerates into Bayes probability theory [63,64]. Some basic concepts of Dempster–Shafer theory are introduced as follows.

**Definition 1** (Frame of discernment)**.** *Assume *Θ* is an exhaustive and finite set of all possible, independent and exclusive values of variable x, indicated by:*

(1) $$\Theta =\left\{x_{1},x_{2},x_{3},\ldots ,x_{n}\right\}$$

*where *Θ* is denoted as a frame of discernment. The power set of *Θ* is $2^{\Theta }$. If $A\in 2^{\Theta }$, then A is called a proposition [65,66,67,68].*

**Definition 2** (Basic probability assignment)**.** *On the frame of discernment *Θ*, assume a mapping m: $2^{\Theta }$ -> [0,1], which satisfies:*

(2) $$m\left(\emptyset \right)=0\;and\;\sum_{A\subseteq \Theta }m\left(A\right)=1$$

*then function m is called mass function or basic probability assignment (BPA).*

In DS evidence theory, m(A) represents how strongly the evidence supports hypothesis *A* [69,70,71]. If m(A)>0, *A* is called a focal element of *m* [72,73].

**Definition 3** (Belief function)**.** *The belief function is a mapping Bel: $2^{\Theta }$ -> [0,1], defined as:*

(3) $$Bel\left(A\right)=\sum_{B\subseteq A}m\left(B\right),\forall A\subseteq \Theta$$

*which represents the total belief on A [74,75]. Belief function is the lower limit function of A.*

**Definition 4** (Plausibility function)**.** *The plausibility function is a mapping Pl: $2^{\Theta }$ -> [0,1], defined as:*

(4) $$Pl\left(A\right)=1-Bel\left(\bar{A}\right)=\sum_{B\cap A\neq \emptyset }m\left(B\right),\forall A\subseteq \Theta$$

*which represents the undeniable degree on A [76,77]. Plausibility function is the upper limit function of A.*

**Definition 5** (DS combination rule)**.** *Assume $m_{1}$ and $m_{2}$ are two independent BPAs on $2^{\Theta }$. In DS evidence theory, their orthogonal sum $m_{1}⊕m_{2}$ is defined as:*

(5) $$m\left(A\right)=\left\{\begin{array}{cc} \frac{1}{1-k}\sum_{B\cap C=A}m_{1}\left(B\right)m_{2}\left(C\right), & A\neq \emptyset .\\ 0, & A=\emptyset . \end{array}\right.$$

*where*

$$k=\sum_{B\cap C=\emptyset }m_{1}\left(B\right)m_{2}\left(C\right)$$

The orthogonal sum in Equation (5) can be extended to the condition when *n* pieces of BPAs are fused simultaneously, which satisfies the mathematical communication law and the associative law. In Equation (5), the constant *k* measures the conflict degree of BPAs [78,79,80]. If *k* is higher, the conflict between BPAs is more serious [81,82,83].

### 2.2. Belief Entropy

In [84], Deng proposed belief entropy. As a generalization of Shannon entropy [85], Deng’s belief entropy can be used to measure the information volume of BPAs. When the belief is only assigned to the single element, Deng’s belief entropy degenerates into Shannon entropy. Many applications have proved the efficiency of Deng entropy [86]. Deng’s belief entropy is defined as follows:

(6) $$E_{d}\left(m\right)=-\sum_{A\subseteq \Theta }m\left(A\right)\log_{2}\frac{m(A)}{2^{\left|A\right|}-1}.$$

where *m* is a BPA defined on the frame of discernment Θ, and *A* is a focal element of *m*. |A| indicates the cardinality of *A*. When the belief is only assigned to single elements of Θ, Deng’s belief entropy degenerates into Shannon entropy, which is shown as:

(7) $$E_{s}\left(m\right)=-\sum_{A\subseteq \Theta }m\left(A\right)\log_{2}m\left(A\right).$$

However, Deng’s belief entropy has some limitations when the propositions are of intersections. To address this shortcoming, Cui et al. [87] improved Deng ’s belief entropy, which takes out the redundant volume created by intersections. Cui et al.’s belief entropy is defined as follows:

(8) $$E_{d}^{*}\left(m\right)=-\sum_{A\subseteq \Theta }m\left(A\right)\log_{2}\left(\frac{m(A)}{2^{\left|A\right|}-1}e^{\sum_{B\subseteq \Theta ,B\neq A}\frac{\left|A\cap B\right|}{2^{\left|\Theta \right|}-1}}\right)$$

where |A| denotes the cardinality of proposition *A*. Here, a numerical example in [87] is used to demonstrate that Cui et al.’s belief entropy is more efficient in measuring the information volume of evidence that contains intersecting propositions.

**Example** **1.** *Assume the frame of discernment is Θ={a,b,c,d}. The values of two BPAs is presented as follows:*

$$m_{1}:m_{1}\left(\left\{a,b\right\}\right)=0.4,m_{1}\left(\left\{c,d\right\}\right)=0.6;$$

$$m_{2}:m_{2}\left(\left\{a,c\right\}\right)=0.4,m_{2}\left(\left\{b,c\right\}\right)=0.6.$$

*According to the data above, both $m_{1}$ and $m_{2}$ have the same scale of focal elements and the same function values. However, the propositions of $m_{2}$ are of intersections, which only contain three elements a, b and c. Therefore, $m_{1}$ has greater information volume than that of $m_{2}$. Next, Deng’s belief entropy and Cui et al.’s belief entropy of two pieces of evidence are calculated as follows.*

*Deng’s belief entropy:*

$$\begin{array}{cc} E_{d}\left(m_{1}\right) & =-\sum_{A\subseteq \Theta }m_{1}\left(A\right)\log_{2}\frac{m_{1}\left(A\right)}{2^{\left|A\right|}-1}\\ & =-0.4\log_{2}\frac{0.4}{2^{2}-1}-0.6\log_{2}\frac{0.6}{2^{2}-1}\\ & =2.5559 \end{array}$$

$$\begin{array}{cc} E_{d}\left(m_{2}\right) & =-\sum_{A\subseteq \Theta }m_{2}\left(A\right)\log_{2}\frac{m_{2}\left(A\right)}{2^{\left|A\right|}-1}\\ & =-0.4\log_{2}\frac{0.4}{2^{2}-1}-0.6\log_{2}\frac{0.6}{2^{2}-1}\\ & =2.5559 \end{array}$$

*Cui et al.’s belief entropy:*

$$\begin{array}{cc} E_{d}^{*}\left(m_{1}\right) & =-\sum_{A\subseteq \Theta }m_{1}\left(A\right)\log_{2}\left(\frac{m_{1}\left(A\right)}{2^{\left|A\right|}-1}e^{\sum_{B\subseteq \Theta ,B\neq A}\frac{\left|A\cap B\right|}{2^{\left|\Theta \right|}-1}}\right)\\ & =-0.4\log_{2}\left(\frac{0.4}{2^{2}-1}e^{0}\right)-0.6\log_{2}\left(\frac{0.6}{2^{2}-1}e^{0}\right)\\ & =2.5559 \end{array}$$

$$\begin{array}{cc} E_{d}^{*}\left(m_{2}\right) & =-\sum_{A\subseteq \Theta }m_{2}\left(A\right)\log_{2}\left(\frac{m_{2}\left(A\right)}{2^{\left|A\right|}-1}e^{\sum_{B\subseteq \Theta ,B\neq A}\frac{\left|A\cap B\right|}{2^{\left|\Theta \right|}-1}}\right)\\ & =-0.4\log_{2}\left(\frac{0.4}{2^{2}-1}e^{\frac{1}{15}}\right)-0.6\log_{2}\left(\frac{0.6}{2^{2}-1}e^{\frac{1}{15}}\right)\\ & =2.4597 \end{array}$$

As shown in Example 1, Deng’s belief entropy ignores the influence of intersections and presents the same uncertain degree of two pieces of evidence. Comparatively, Cui et al.’s belief entropy takes the redundant space of intersections out from their information volume. Therefore, if the evidence has many intersecting propositions, it would be better to use Cui et al.’s belief entropy to measure their information volume more accurately. Besides, if the evidence has greater belief entropy, it contains more information and there fewer less conflicts between this subset and the frame of discernment. Therefore, the evidence that has greater belief entropy should be assigned more weights in the fusing procedure.

### 2.3. Belief Jenson–Shannon Divergence

In [88], Xiao proposed Belief Jensen–Shannon (BJS) divergence by integrating DS evidence theory and Jenson–Shannon divergence. Suppose $m_{1}$ and $m_{2}$ are two BPAs on the same frame of discernment that contains *n* elements; the BJS divergence between $m_{1}$ and $m_{2}$ is defined as:

(9) $$BJS\left(m_{1},m_{2}\right)=\frac{1}{2}\left[S\left(m_{1},\frac{m_{1}+m_{2}}{2}\right)+S\left(m_{2},\frac{m_{1}+m_{2}}{2}\right)\right],$$

where

(10) $$S\left(m_{1},m_{2}\right)=\sum_{i}^{n}m_{1}\left(A_{i}\right)\log\frac{m_{1}\left(A_{i}\right)}{m_{2}\left(A_{i}\right)}.$$

The main contribution of the belief Jenson–Shannon divergence is that it replaces the probabilities distributions in JS divergence with BPAs, so that BJS divergence can be applied in DS evidence theory to measure the difference between BPAs.

## 3. The Proposed Method

**Figure 1:**
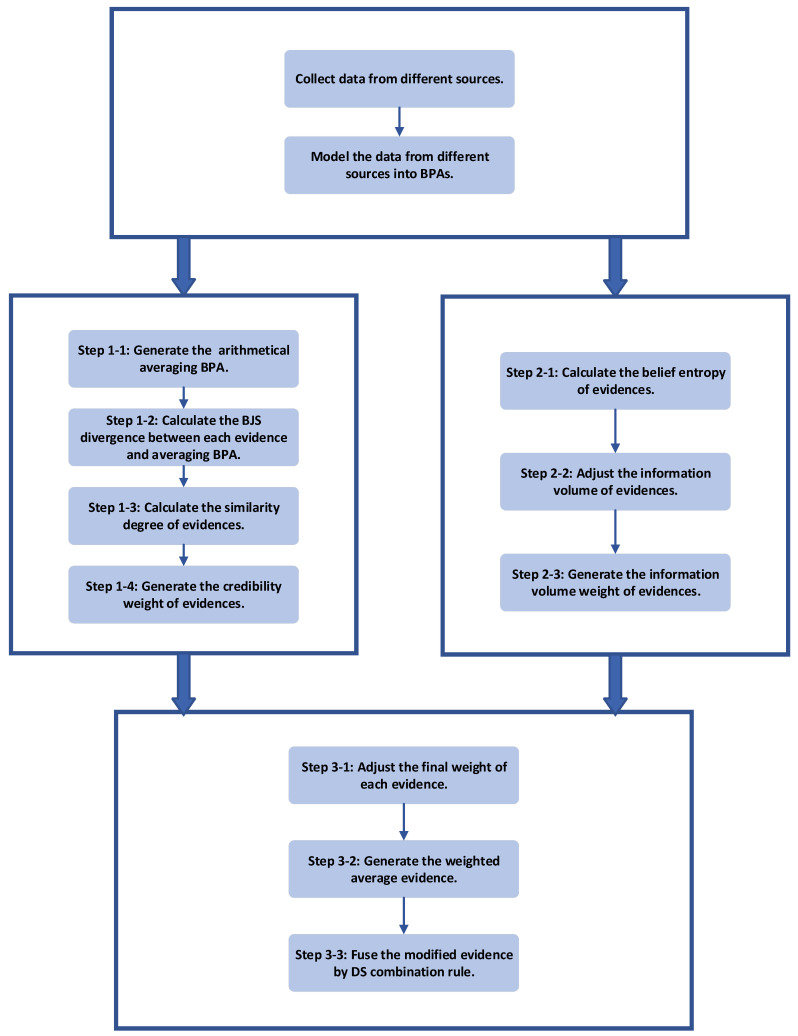
The flowchart of the proposed method.

To address the problem of fusing highly-conflicted evidence, a new combination method is proposed in this section. To allocate the weight on evidence more properly, not only credibility of the evidence but also its information volume is taken into consideration. The procedure is divided into three parts. Firstly, the credibility weight of evidence is obtained after transforming BJS divergence into similarities. Secondly, the information volume weight of evidence is obtained by calculating the belief entropy. Thirdly, the weighted average BPA is generated before fusing it by DS combination rule. The flowchart of the method is shown in Figure 1. More details and explanations about each step of the method are described as follows.

### 3.1. Calculate the Credibility Weight of Evidences

**Step 1-1.** Suppose $M=\{m_{1},m_{2},\ldots ,m_{n}\}$ is a set of *n* independent BPAs on the same frame of discernment that contains *N* elements: $\Theta =\{F_{1},F_{2},F_{3},\ldots ,F_{N}\}$. The arithmetical average BPA $m_{a}$ is defined as:

(11) $$m_{a}\left(A\right)=\frac{1}{n}\sum_{i=1}^{n}m_{i}\left(A\right),\forall A\subseteq \Theta .$$

**Step 1-2.** Calculate the BJS divergence between $m_{i}$ and $m_{a}$(i=1,2,3,…,n) according to Equation (9).

(12) $$BJS\left(m_{i},m_{a}\right)=\frac{1}{2}\left[S\left(m_{i},\frac{m_{i}+m_{a}}{2}\right)+S\left(m_{a},\frac{m_{i}+m_{a}}{2}\right)\right],$$

where

$$S\left(m_{1},m_{2}\right)=\sum_{i}^{n}m_{1}\left(A_{i}\right)\log\frac{m_{1}\left(A_{i}\right)}{m_{2}\left(A_{i}\right)}.$$

**Step 1-3.** Since the similarities of the pieces of evidence are negatively correlated with their divergences, if the divergence between two pieces of evidence is higher, they have lower similarity. The divergence between $m_{i}$ and $m_{a}$ is converted into their similarity as follows:

(13) $$Sim\left(m_{i},m_{a}\right)=e^{-BJS(m_{i},m_{a})},i=1,2,3,\ldots ,n.$$

**Step 1-4.** If a piece of evidence is highly similar to the average BPA, it means that the evidence is supported by most of the other pieces of evidence and it is more reliable, thus it gains high credibility. Thus, the credibility weight of the pieces of evidence is determined by normalizing their similarity with the arithmetical average BPA. The credibility weight ($W_{c}$) of each piece of evidence is defined as follows:

(14) $$W_{c}\left(m_{i}\right)=\frac{Sim(m_{i},m_{a})}{\sum_{i}Sim\left(m_{i},m_{a}\right)},i=1,2,3,\ldots ,n$$

### 3.2. Calculate Information Volume Weight of Evidence

**Step 2-1.** Calculate the belief entropy of $m_{i}$ (i=1,2,3,…,n) according to Equation (8).

(15) $$E_{d}^{*}\left(m_{i}\right)=-\sum_{A\subseteq \Theta }m_{i}\left(A\right)\log_{2}\left(\frac{m_{i}\left(A\right)}{2^{\left|A\right|}-1}e^{\sum_{B\subseteq \Theta ,B\neq A}\frac{\left|A\cap B\right|}{2^{\left|\Theta \right|}-1}}\right),$$

**Step 2-2.** To avoid assigning zero weight to the evidence whose belief entropy is zero, the information volume in Step 2-1 is modified as follows:

(16) $$IV_{i}=e^{E_{d}^{*}\left(m_{i}\right)},i=1,2,3,\ldots ,n$$

where $E_{d}^{*}\left(m_{i}\right)$ represents the belief entropy of $m_{i}$.

**Step 2-3.** Calculate the information volume weight ($W_{iv}$) of each piece of evidence by normalizing IV, which is defined as:

(17) $$W_{iv}\left(m_{i}\right)=\frac{IV_{i}}{\sum_{i}IV_{i}},i=1,2,3,\ldots ,n.$$

### 3.3. Generate the Modified Evidence and Fuse

**Step 3-1.** Based on the credibility weight and information volume weight of evidence, the weight of each piece of evidence is adjusted as follows:

(18) $$W\left(m_{i}\right)=W_{c}\left(m_{i}\right)\times W_{iv}\left(m_{i}\right),i=1,2,3,\ldots ,n.$$

**Step 3-2.** Normalize the modified weight as follows:

(19) $$W^{*}\left(m_{i}\right)=\frac{W(m_{i})}{\sum_{i}W\left(m_{i}\right)},i=1,2,3,\ldots ,n.$$

**Step 3-3.** Generate the modified evidence by calculating the weighted average sum of BPAs, which is defined as:

(20) $$m^{*}\left(A\right)=\sum_{i=1}^{n}W^{*}\left(m_{i}\right)\times m_{i}\left(A\right),\forall A\subseteq \Theta .$$

**Step 3-4.** DS combination rule is used n−1 times on the modified evidence based on [52], then the final combination result is obtained:

(21) $$\begin{array}{cc} m_{⊕}\left(A\right) & ={\left(m^{*}\left(A\right)⊕m^{*}\left(A\right)⊕m^{*}\left(A\right)\ldots m^{*}\left(A\right)\right)}_{n-1}\\ & ={\left({\left({\left(m^{*}\left(A\right)⊕m^{*}\left(A\right)\right)}_{1}⊕m^{*}\left(A\right)\right)}_{2}\ldots ⊕m^{*}\left(A\right)\right)}_{n-1} \end{array}$$

## 4. Numerical Example

In this section, the proposed method is compared with other existing combination rules by a numerical example in [55] and the effectiveness of the proposed method is illustrated.

### 4.1. Example Presentation

Assume the frame of discernment Θ={A,B,C}. There are five pieces of evidence denoted as $m_{1}$, $m_{2}$, $m_{3}$, $m_{4}$, and $m_{5}$ and their mass functions are listed in Table 1. Here, evidence $m_{2}$ is not credible as other pieces of evidence since the sensor which is modeled into $m_{2}$ is disturbed by some unknown environmental factors.

### 4.2. Combination by the Proposed Method

**Step 1-1.** Calculate the arithmetical average BPA.

$$\begin{array}{c} m_{a}\left(A\right)=0.4280\\ m_{a}\left(B\right)=0.2920\\ m_{a}\left(C\right)=0.0800\\ m_{a}\left(\left\{A,C\right\}\right)=0.2000 \end{array}$$

**Step 1-2.** Calculate the BJS divergence between each piece of evidence and $m_{a}$.

$$\begin{array}{c} BJS(m_{1},m_{a})=0.1033\\ BJS(m_{2},m_{a})=0.2995\\ BJS(m_{3},m_{a})=0.0804\\ BJS(m_{4},m_{a})=0.0655\\ BJS(m_{5},m_{a})=0.0645 \end{array}$$

**Step 1-3.** Calculate the similarity degree of each piece of evidence.

$$\begin{array}{c} Sim(m_{1},m_{a})=0.9018\\ Sim(m_{2},m_{a})=0.7412\\ Sim(m_{3},m_{a})=0.9227\\ Sim(m_{4},m_{a})=0.9357\\ Sim(m_{5},m_{a})=0.9375 \end{array}$$

**Step 1-4.** Calculate the weight of credibility.

$$\begin{array}{c} W_{c}\left(m_{1}\right)=0.2032\\ W_{c}\left(m_{2}\right)=0.1670\\ W_{c}\left(m_{3}\right)=0.2079\\ W_{c}\left(m_{4}\right)=0.2108\\ W_{c}\left(m_{5}\right)=0.2112 \end{array}$$

**Step 2-1.** Calculate the belief entropy of each piece of evidence.

$$\begin{array}{c} E_{d}^{*}\left(m_{1}\right)=1.3603\\ E_{d}^{*}\left(m_{2}\right)=0.2629\\ E_{d}^{*}\left(m_{3}\right)=1.5310\\ E_{d}^{*}\left(m_{4}\right)=1.6132\\ E_{d}^{*}\left(m_{5}\right)=1.5030 \end{array}$$

**Step 2-2.** Adjust the information volume of each piece of evidence.

$$\begin{array}{c} IV_{1}=3.8973\\ IV_{2}=1.3007\\ IV_{3}=4.6226\\ IV_{4}=5.0187\\ IV_{5}=4.4953 \end{array}$$

**Step 2-3.** Calculate the weight of information volume.

$$\begin{array}{c} W_{iv}\left(m_{1}\right)=0.2016\\ W_{iv}\left(m_{2}\right)=0.0673\\ W_{iv}\left(m_{3}\right)=0.2391\\ W_{iv}\left(m_{4}\right)=0.2596\\ W_{iv}\left(m_{5}\right)=0.2325 \end{array}$$

**Step 3-1.** Adjust the weight of each piece of evidence.

$$\begin{array}{c} W(m_{1})=0.0410\\ W(m_{2})=0.0112\\ W(m_{3})=0.0497\\ W(m_{4})=0.0547\\ W(m_{5})=0.0491 \end{array}$$

**Step 3-2.** Normalize the modified weight of each piece of evidence.

$$\begin{array}{c} W^{*}\left(m_{1}\right)=0.1991\\ W^{*}\left(m_{2}\right)=0.0546\\ W^{*}\left(m_{3}\right)=0.2416\\ W^{*}\left(m_{4}\right)=0.2660\\ W^{*}\left(m_{5}\right)=0.2387 \end{array}$$

**Step 3-3.** Generate the modified evidence.

$$\begin{array}{c} m^{*}\left(\left\{A\right\}\right)=0.5113\\ m^{*}\left(\left\{B\right\}\right)=0.1743\\ m^{*}\left(\left\{C\right\}\right)=0.0652\\ m^{*}\left(\left\{A,C\right\}\right)=0.2082 \end{array}$$

**Step 3-4.** Use the classical DS combination rule to fuse the modified evidence four times, and the result is shown in Table 2.

### 4.3. Analysis

**Figure 2:**
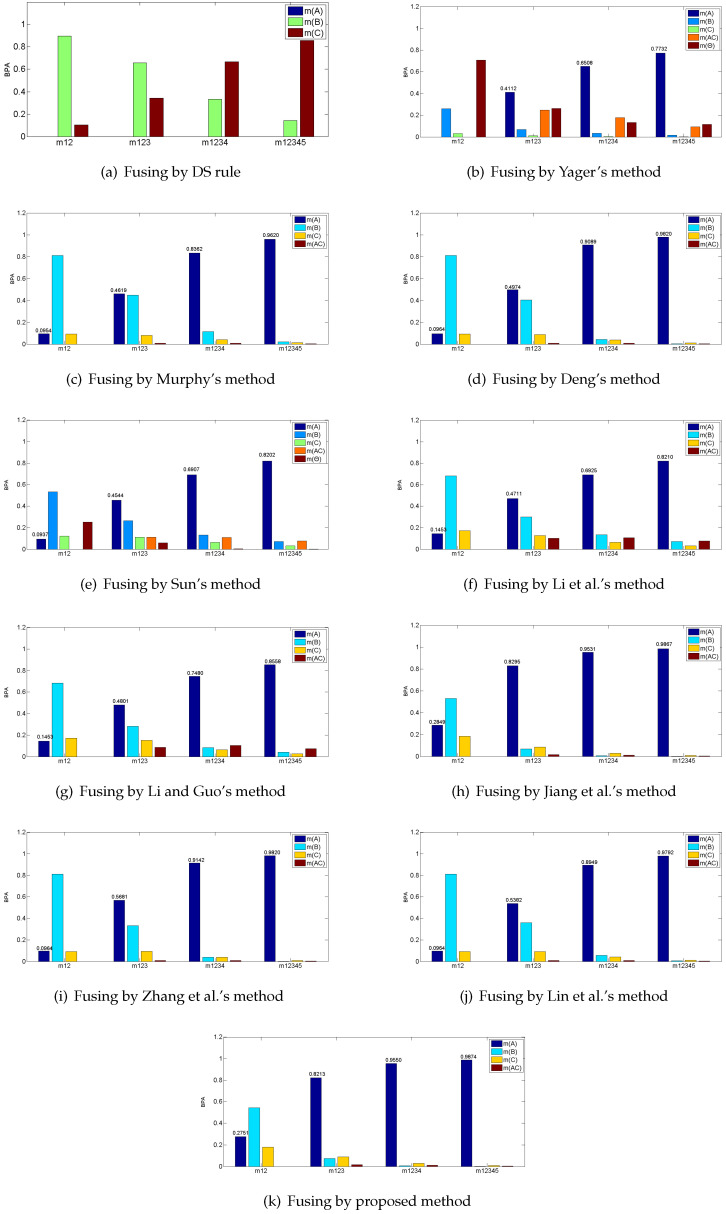
Fusing results by different combination methods in the example.

According to Table 1, $m_{2}$ shows great conflict with the four other pieces of evidence, which assigns most of the belief on *B*, while the remaining four pieces of evidence all support *A*. In this case, the major belief after fusing should be allocated on *A* since the $m_{2}$ is modeled from an abnormal sensor. The fusing results of the proposed method and other existing combination rules are presented in Table 2 and Figure 2.

As shown in Table 2, classical DS combination rule is disturbed by the abnormal evidence and assigns most of its belief on *C* wrongly. The remaining combination rules all present the reasonable results and majorly support *A*. The incredible evidence $m_{2}$ appears on the first time fusion and misguides the fusing process to recognize *C*, but as the later credible pieces of evidence join the fusion process, these combination methods all turn to assign their beliefs mainly on *A*. However, in the real situation, a slight increase of accuracy is significant to improve the performance of the system [54,55,56]. Comparatively, the proposed method achieves the highest accuracy of 0.9874 for identifying *A* among these combination rules. Therefore, the proposed method is relatively effective because it takes two important factors of evidence—the credibility and information volume—into consideration, so that the weight of the incredible evidence is controlled more strictly.

## 5. Application

### 5.1. Problem Statement

An automobile system was excessively used and it caused shortage of power. According to the records in the database, there were three possible faults that may lead to this problem: low oil pressure, air leakage in the intake system and a stuck solenoid valve, which are denoted as $F_{1},F_{2}$ and $F_{3}$, respectively. Five sensors, denoted as $S_{1},S_{2},S_{3},S_{4}$ and $S_{5}$, were placed at different positions to measure the parameters including the air displacement, maximum power, maximum torsion, compression ratio and maximum rotation speed of the power system. After all the sensors finished measuring, the central controlling system then modeled the parameters detected from sensors to BPAs, which are presented in Table 3, where $\Theta =\{F_{1},F_{2},F_{3}\}$ is the frame of discernment and $m_{i}$ is the evidence modeling from $S_{i}$ (i=1,2,3,4,5). In this application, $S_{5}$ was broken because the engine speed accidentally exceeded its upper limitation and could not function normally as the four other sensors. The objective is to judge which type of fault has occurred in the automobile system according to these pieces of evidence.

### 5.2. Fuse Evidences by the Proposed Method

**Step 1-1.** Calculate the arithmetical average BPA.

$$\begin{array}{c} m_{a}\left(F_{1}\right)=0.5600\\ m_{a}\left(F_{2}\right)=0.0900\\ m_{a}\left(F_{3}\right)=0.1700\\ m_{a}\left(\Theta \right)=0.1800 \end{array}$$

**Step 1-2.** Calculate the BJS divergence between each piece of evidence and $m_{a}$.

$$\begin{array}{c} BJS(m_{1},m_{a})=0.0632\\ BJS(m_{2},m_{a})=0.1016\\ BJS(m_{3},m_{a})=0.0646\\ BJS(m_{4},m_{a})=0.0557\\ BJS(m_{5},m_{a})=0.3782 \end{array}$$

**Step 1-3.** Calculate the similarity degree of each piece of evidence.

$$\begin{array}{c} Sim(m_{1},m_{a})=0.9387\\ Sim(m_{2},m_{a})=0.9034\\ Sim(m_{3},m_{a})=0.9374\\ Sim(m_{4},m_{a})=0.9459\\ Sim(m_{5},m_{a})=0.6851 \end{array}$$

**Step 1-4.** Calculate the weight of credibility.

$$\begin{array}{c} W_{c}\left(m_{1}\right)=0.2128\\ W_{c}\left(m_{2}\right)=0.2048\\ W_{c}\left(m_{3}\right)=0.2125\\ W_{c}\left(m_{4}\right)=0.2145\\ W_{c}\left(m_{5}\right)=0.1553 \end{array}$$

**Step 2-1.** Calculate the belief entropy of each piece of evidence.

$$\begin{array}{c} E_{d}^{*}\left(m_{1}\right)=1.4297\\ E_{d}^{*}\left(m_{2}\right)=1.3937\\ E_{d}^{*}\left(m_{3}\right)=1.5518\\ E_{d}^{*}\left(m_{4}\right)=1.2647\\ E_{d}^{*}\left(m_{5}\right)=0.5158 \end{array}$$

**Step 2-2.** Adjust the information volume of each piece of evidence.

$$\begin{array}{c} IV_{1}=4.1775\\ IV_{2}=4.0299\\ IV_{3}=4.7201\\ IV_{4}=3.5420\\ IV_{5}=1.6750 \end{array}$$

**Step 2-3.** Calculate the weight of information volume.

$$\begin{array}{c} W_{iv}\left(m_{1}\right)=0.2302\\ W_{iv}\left(m_{2}\right)=0.2221\\ W_{iv}\left(m_{3}\right)=0.2601\\ W_{iv}\left(m_{4}\right)=0.1952\\ W_{iv}\left(m_{5}\right)=0.0923 \end{array}$$

**Step 3-1.** Adjust the weight of each piece of evidence.

$$\begin{array}{c} W(m_{1})=0.0490\\ W(m_{2})=0.0455\\ W(m_{3})=0.0553\\ W(m_{4})=0.0419\\ W(m_{5})=0.0143 \end{array}$$

**Step 3-2.** Normalize the modified weight of each piece of evidence.

$$\begin{array}{c} W^{*}\left(m_{1}\right)=0.2379\\ W^{*}\left(m_{2}\right)=0.2208\\ W^{*}\left(m_{3}\right)=0.2683\\ W^{*}\left(m_{4}\right)=0.2033\\ W^{*}\left(m_{5}\right)=0.0696 \end{array}$$

**Step 3-3.** Generate the modified evidence.

$$\begin{array}{c} m^{*}\left(F_{1}\right)=0.6480\\ m^{*}\left(F_{2}\right)=0.0780\\ m^{*}\left(F_{3}\right)=0.0659\\ m^{*}\left(\Theta \right)=0.2082 \end{array}$$

**Step 3-4.** Use the classical DS combination rule to fuse the modified evidence four times, and the result is shown in Table 4.

### 5.3. Discussion

**Figure 3:**
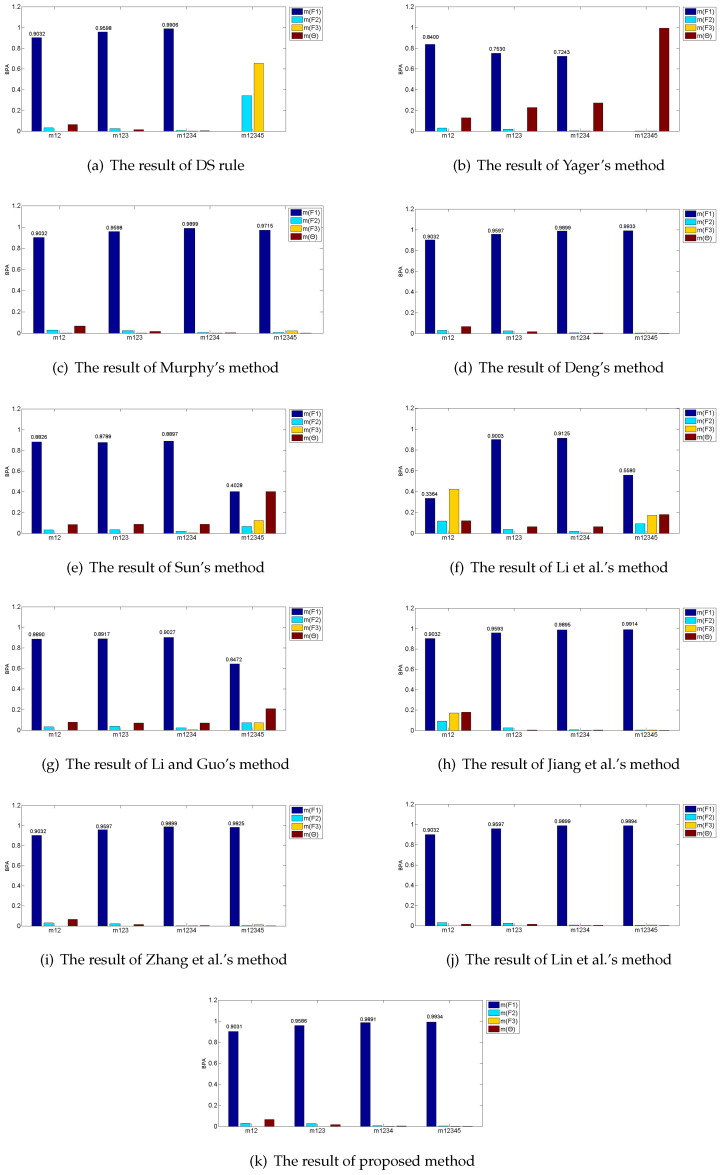
Fusing results by different combination methods in the application.

Table 4 and Figure 3 show the results of fusing five BPAs by applying the proposed method, together with the other existing combination rules. Here, a threshold △=0.70 is set based on [54]. After fusing by the combination method, if $m(F_{i})\geq 0.70$, it means that the method recognizes fault $F_{i}$ successfully; otherwise, this combination rule cannot identify what kind of fault and “unknown” is marked in Table 4.

In this application, $S_{1}$, $S_{2}$, $S_{3}$, and $S_{4}$ all work well, and the pieces of evidence after transforming their detected data are greatly consistent, which all assign most of their beliefs to $F_{1}$ according to Table 3. However, $S_{5}$ is broken and the evidence after modeling the data collected from $S_{5}$ intensively conflicts with the other four pieces of evidence, which assigns most of the belief to $F_{3}$ wrongly. Based on these considerations, $F_{1}$ is judged as the fault in the automobile system. The ideal result after fusing by these combination methods is to recognize fault $F_{1}$ accurately, ignoring the disturbing effect of $S_{5}$.

As shown in Table 4, the classical DS combination rule successfully recognizes fault $F_{1}$ after fusing the first four pieces of evidence, but, when the incredible evidence $m_{5}$ joins, it drastically reassigns most of the beliefs to the fault $F_{3}$ wrongly. Therefore, classical DS combination rule fails to fuse the highly conflicting evidence. Yager’s method reallocates the conflicting degree to the unknown space and it cannot identify which type of fault. Sun et al.’s method, Li and Guo’s method and Li et al.’s method do not reach the threshold △=0.70. As a result, they cannot distinguish the targeted type of fault. Deng’s method, Jiang’s method, Zhang’s method, Lin et al.’s method and the proposed method all recognize the fault $F_{1}$ successfully. Deng introduced a combination method based on the distances among evidences and it presents 99.33% recognition accuracy comparing with 99.34% of the proposed method, which indicates that Deng’s method can deal with the conflicting evidence. However, in Deng’s method, the sizes of its distance matrix and similarity matrix are both n×n, while the sizes of divergence vector and information volume vector are both 1×n in the proposed method. Therefore, the proposed method presents better accuracy of targeted type of fault with a lower computational complexity algorithm comparing with Deng’s method. It is worth noting that a slight increase of accuracy is significant to improve the performance of the system in the real application [54,55,56]. When the conflicting evidence $m_{5}$ joins the fusion, Lin et al.’s method shows a slight decrease from 98.99% to 98.94% and Jiang et al.’s method climbs from 98.95% to 99.14%, while the proposed method increases from 98.91% to 99.34%. This means that the proposed method can overcome the influence of incredible evidence better and maintain the degree of increase as more pieces of evidence join. Overall, the proposed method can diagnosis the targeted type of fault more accurately than other combination rules as it assigns the highest belief of 0.9934 to the fault $F_{1}$. The reason is that the proposed method not only makes use of Belief Jenson–Shannon function to measure the credibility of the evidence, but also considers their information volume by applying belief entropy before allocating the final weight to each piece of evidence. Since the conflicted evidence $m_{5}$ has not only low credibility but small information volume, its weight is declined, so that the influence of fault evidence is controlled more strictly in comparison with the other proposed combination rules.

## 6. Conclusions

Dempster–Shafer evidence theory is widely used in information fusion field due to its efficiency in handling uncertainty and imprecision. However, some counter-intuitive results occur when the evidence is highly in conflict. To address this shortcoming, a novel multi-source combination method is proposed in this paper based on BJS divergence measure and the improved Deng entropy. Not only the credibility but also the information volume of the evidence is taken into consideration to allocate the weight on the original evidence before fusing the modified evidence, so that the influence of untruthful evidence is controlled more seriously, resulting in the higher attention on the credible evidence when fusing. Next, the proposed method is compared with other existing combination rules in a numerical example. The result shows that the proposed method achieves the highest accuracy of 99.74% among these combination rules. Furthermore, the proposed method is applied in an application of fault diagnosis to identify the type of fault in an automobile system. Among the combination rules which successfully recognized the correct type of fault, the proposed method shows the highest degree of accuracy of 99.34% with lower computational complexity since the sizes of two vectors are both 1×n rather than n×n. In summary, this study provides a promising way to deal with the multi-source data fusion problems. In the near future, to make the proposed method more applicable in the real environment, how to generate BPAs more properly from different sources will be further considered.

## Figures and Tables

**Table 1:** A numerical example in [55].

	{A}	{B}	{C}	{A,C}
$m_{1}$	0.41	0.29	0.30	0
$m_{2}$	0	0.90	0.10	0
$m_{3}$	0.58	0.07	0	0.35
$m_{4}$	0.55	0.10	0	0.35
$m_{5}$	0.60	0.10	0	0.30

**Table 2:** Fusing results by different methods in the example.

Method	$m_{1,2}$	$m_{1,2,3}$	$m_{1,2,3,4}$	$m_{1,2,3,4,5}$
DS [38]	m(A)=0.0000 m(B)=0.8969 m(C)=0.1031	m(A)=0.0000 m(B)=0.6575 m(C)=0.3425	m(A)=0.0000 m(B)=0.3321 m(C)=0.6679	m(A)=0.0000 m(B)=0.1422 m(C)=0.8578
Yager [47]	m(A)=0.0000 m(B)=0.2610 m(C)=0.0300 m(A,C)=0.0000 m(Θ)=0.7090	m(A)=0.4112 m(B)=0.0679 m(C)=0.0105 m(A,C)=0.2481 m(Θ)=0.2622	m(A)=0.6508 m(B)=0.0330 m(C)=0.0037 m(A,C)=0.1786 m(Θ)=0.1339	m(A)=0.7732 m(B)=0.0167 m(C)=0.0011 m(A,C)=0.0938 m(Θ)=0.1152
Murphy [52]	m(A)=0.0964 m(B)=0.8119 m(C)=0.0917 m(A,C)=0.0000	m(A)=0.4619 m(B)=0.4497 m(C)=0.0794 m(A,C)=0.0090	m(A)=0.8362 m(B)=0.1147 m(C)=0.0410 m(A,C)=0.0081	m(A)=0.9620 m(B)=0.0210 m(C)=0.0138 m(A,C)=0.0032
Deng et al. [53]	m(A)=0.0964 m(B)=0.8119 m(C)=0.0917 m(A,C)=0.0000	m(A)=0.4974 m(B)=0.4054 m(C)=0.0888 m(A,C)=0.0084	m(A)=0.9089 m(B)=0.0444 m(C)=0.0379 m(A,C)=0.0089	m(A)=0.9820 m(B)=0.0039 m(C)=0.0107 m(A,C)=0.0034
Sun et al. [50]	m(A)=0.0937 m(B)=0.5330 m(C)=0.1214 m(A,C)=0.0000 m(Θ)=0.2519	m(A)=0.4544 m(B)=0.2639 m(C)=0.1107 m(A,C)=0.1124 m(Θ)=0.0585	m(A)=0.6907 m(B)=0.1324 m(C)=0.0636 m(A,C)=0.1088 m(Θ)=0.0045	m(A)=0.8202 m(B)=0.0711 m(C)=0.0309 m(A,C)=0.0760 m(Θ)=0.0018
Li et al. [51]	m(A)=0.1453 m(B)=0.6829 m(C)=0.1718 m(A,C)=0.0000	m(A)=0.4711 m(B)=0.3007 m(C)=0.1266 m(A,C)=0.1016	m(A)=0.6925 m(B)=0.1350 m(C)=0.0650 m(A,C)=0.1074	m(A)=0.8210 m(B)=0.0719 m(C)=0.0314 m(A,C)=0.0757
Li and Guo [89]	m(A)=0.1453 m(B)=0.6829 m(C)=0.1718 m(A,C)=0.0000	m(A)=0.4801 m(B)=0.2821 m(C)=0.1512 m(A,C)=0.0866	m(A)=0.7480 m(B)=0.0850 m(C)=0.0630 m(A,C)=0.1040	m(A)=0.8558 m(B)=0.0425 m(C)=0.0267 m(A,C)=0.0750
Jiang et al. [54]	m(A)=0.2849 m(B)=0.5306 m(C)=0.1845 m(A,C)=0.0000	m(A)=0.8295 m(B)=0.0680 m(C)=0.0854 m(A,C)=0.0171	m(A)=0.9531 m(B)=0.0074 m(C)=0.0292 m(A,C)=0.0103	m(A)=0.9867 m(B)=0.0008 m(C)=0.0089 m(A,C)=0.0036
Zhang et al. [55]	m(A)=0.0964 m(B)=0.8119 m(C)=0.0917 m(A,C)=0.0000	m(A)=0.5681 m(B)=0.3319 m(C)=0.0929 m(A,C)=0.0084	m(A)=0.9142 m(B)=0.0395 m(C)=0.0399 m(A,C)=0.0083	m(A)=0.9820 m(B)=0.0034 m(C)=0.0115 m(A,C)=0.0032
Lin et al. [56]	m(A)=0.0964 m(B)=0.8119 m(C)=0.0917 m(A,C)=0.0000	m(A)=0.5382 m(B)=0.3599 m(C)=0.0927 m(A,C)=0.0076	m(A)=0.8949 m(B)=0.0558 m(C)=0.0413 m(A,C)=0.0080	m(A)=0.9792 m(B)=0.0057 m(C)=0.0119 m(A,C)=0.0032
Proposed method	m(A)=0.2751 m(B)=0.5446 m(C)=0.1803 m(A,C)=0.0000	m(A)=0.8213 m(B)=0.0738 m(C)=0.0890 m(A,C)=0.0159	m(A)=0.9550 m(B)=0.0063 m(C)=0.0281 m(A,C)=0.0106	m(A)=0.9874 m(B)=0.0006 m(C)=0.0082 m(A,C)=0.0037

**Table 3:** BPAs after modeling from sensors.

	$F_{1}$	$F_{2}$	$F_{3}$	Θ
$m_{1}$	0.70	0.10	0	0.20
$m_{2}$	0.70	0	0	0.30
$m_{3}$	0.65	0.15	0	0.20
$m_{4}$	0.75	0	0.05	0.20
$m_{5}$	0	0.20	0.80	0

**Table 4:** Fusing results of the application.

Method	$m_{1,2}$	$m_{1,2,3}$	$m_{1,2,3,4}$	$m_{1,2,3,4,5}$	Recognized Fault
DS [38]	$m(F_{1})=0.9032$ $m(F_{2})=0.0323$ $m(F_{3})=0.0000$ m(Θ)=0.0645	$m(F_{1})=0.9598$ $m(F_{2})=0.0249$ $m(F_{3})=0.0000$ m(Θ)=0.0153	$m(F_{1})=0.9906$ $m(F_{2})=0.0053$ $m(F_{3})=0.0008$ m(Θ)=0.0033	$m(F_{1})=0.0000$ $m(F_{2})=0.3443$ $m(F_{3})=0.6557$ m(Θ)=0.0000	Unknown
Yager [47]	$m(F_{1})=0.8400$ $m(F_{2})=0.0300$ $m(F_{3})=0.0000$ m(Θ)=0.1300	$m(F_{1})=0.7530$ $m(F_{2})=0.0195$ $m(F_{3})=0.0000$ m(Θ)=0.2275	$m(F_{1})=0.7243$ $m(F_{2})=0.0039$ $m(F_{3})=0.0006$ m(Θ)=0.2721	$m(F_{1})=0.0000$ $m(F_{2})=0.0013$ $m(F_{3})=0.0024$ m(Θ)=0.9963	Unknown
Murphy [52]	$m(F_{1})=0.9032$ $m(F_{2})=0.0296$ $m(F_{3})=0.0000$ m(Θ)=0.0672	$m(F_{1})=0.9598$ $m(F_{2})=0.0241$ $m(F_{3})=0.0000$ m(Θ)=0.0161	$m(F_{1})=0.9899$ $m(F_{2})=0.0058$ $m(F_{3})=0.0008$ m(Θ)=0.0035	$m(F_{1})=0.9715$ $m(F_{2})=0.0055$ $m(F_{3})=0.0222$ m(Θ)=0.0008	$F_{1}$
Deng et al. [53]	$m(F_{1})=0.9032$ $m(F_{2})=0.0296$ $m(F_{3})=0.0000$ m(Θ)=0.0672	$m(F_{1})=0.9597$ $m(F_{2})=0.0243$ $m(F_{3})=0.0000$ m(Θ)=0.0160	$m(F_{1})=0.9899$ $m(F_{2})=0.0058$ $m(F_{3})=0.0008$ m(Θ)=0.0035	$m(F_{1})=0.9933$ $m(F_{2})=0.0030$ $m(F_{3})=0.0028$ m(Θ)=0.0008	$F_{1}$
Sun et al. [50]	$m(F_{1})=0.8826$ $m(F_{2})=0.0330$ $m(F_{3})=0.0000$ m(Θ)=0.0844	$m(F_{1})=0.8789$ $m(F_{2})=0.0348$ $m(F_{3})=0.0000$ m(Θ)=0.0863	$m(F_{1})=0.8897$ $m(F_{2})=0.0187$ $m(F_{3})=0.0036$ m(Θ)=0.0880	$m(F_{1})=0.4028$ $m(F_{2})=0.0660$ $m(F_{3})=0.1247$ m(Θ)=0.4028	Unknown
Li et al. [51]	$m(F_{1})=0.3364$ $m(F_{2})=0.1185$ $m(F_{3})=0.4250$ m(Θ)=0.1201	$m(F_{1})=0.9003$ $m(F_{2})=0.0375$ $m(F_{3})=0.0000$ m(Θ)=0.0623	$m(F_{1})=0.9125$ $m(F_{2})=0.0207$ $m(F_{3})=0.0040$ m(Θ)=0.0629	$m(F_{1})=0.5580$ $m(F_{2})=0.0910$ $m(F_{3})=0.1718$ m(Θ)=0.1793	Unknown
Li and Guo [89]	$m(F_{1})=0.8890$ $m(F_{2})=0.0335$ $m(F_{3})=0.0000$ m(Θ)=0.0775	$m(F_{1})=0.8917$ $m(F_{2})=0.0394$ $m(F_{3})=0.0000$ m(Θ)=0.0689	$m(F_{1})=0.9027$ $m(F_{2})=0.0226$ $m(F_{3})=0.0043$ m(Θ)=0.0704	$m(F_{1})=0.6472$ $m(F_{2})=0.0732$ $m(F_{3})=0.0717$ m(Θ)=0.2079	Unknown
Jiang et al. [54]	$m(F_{1})=0.9032$ $m(F_{2})=0.0910$ $m(F_{3})=0.1718$ m(Θ)=0.1793	$m(F_{1})=0.9593$ $m(F_{2})=0.0247$ $m(F_{3})=0.0000$ m(Θ)=0.0035	$m(F_{1})=0.9895$ $m(F_{2})=0.0062$ $m(F_{3})=0.0008$ m(Θ)=0.0035	$m(F_{1})=0.9914$ $m(F_{2})=0.0035$ $m(F_{3})=0.0042$ m(Θ)=0.0009	$F_{1}$
Zhang et al. [55]	$m(F_{1})=0.9032$ $m(F_{2})=0.0296$ $m(F_{3})=0.0000$ m(Θ)=0.0672	$m(F_{1})=0.9597$ $m(F_{2})=0.0242$ $m(F_{3})=0.0000$ m(Θ)=0.0161	$m(F_{1})=0.9899$ $m(F_{2})=0.0058$ $m(F_{3})=0.0008$ m(Θ)=0.0035	$m(F_{1})=0.9825$ $m(F_{2})=0.0045$ $m(F_{3})=0.0122$ m(Θ)=0.0008	$F_{1}$
Lin et al. [56]	$m(F_{1})=0.9032$ $m(F_{2})=0.0296$ $m(F_{3})=0.0000$ m(Θ)=0.0159	$m(F_{1})=0.9597$ $m(F_{2})=0.0244$ $m(F_{3})=0.0000$ m(Θ)=0.0159	$m(F_{1})=0.9899$ $m(F_{2})=0.0058$ $m(F_{3})=0.0008$ m(Θ)=0.0035	$m(F_{1})=0.9894$ $m(F_{2})=0.0037$ $m(F_{3})=0.0061$ m(Θ)=0.0008	$F_{1}$
Proposed method	$m(F_{1})=0.9031$ $m(F_{2})=0.0303$ $m(F_{3})=0.0000$ m(Θ)=0.0667	$m(F_{1})=0.9586$ $m(F_{2})=0.0257$ $m(F_{3})=0.0000$ m(Θ)=0.0157	$m(F_{1})=0.9891$ $m(F_{2})=0.0061$ $m(F_{3})=0.0007$ m(Θ)=0.0035	$m(F_{1})=0.9934$ $m(F_{2})=0.0033$ $m(F_{3})=0.0025$ m(Θ)=0.0008	$F_{1}$

## References

[1] Alam F., Mehmood R., Katib I., Albogami N.N., Albeshri A. (2017). Data Fusion and IoT for Smart Ubiquitous Environments: A Survey. IEEE Access.

[2] Knuth K.H., Shah A.S., Truccolo W.A., Mingzhou D., Bressler S.L., Schroeder C.E. (2013). Differentially variable component analysis: Identifying multiple evoked components using trial-to-trial variability. J. Neurophysiol..

[3] Zhou D., Al-Durra A., Gao F., Ravey A., Matraji I., Simoes M.G. (2017). Online energy management strategy of fuel cell hybrid electric vehicles based on data fusion approach. J. Power Sources.

[4] Zhou D., Al-Durra A., Zhang K., Ravey A., Gao F. (2019). A Robust Prognostic Indicator for Renewable Energy Technologies: A Novel Error Correction Grey Prediction Model. IEEE Trans. Ind. Electron..

[5] Cao Z., Lai K.L., Lin C.T., Chuang C.H., Chou C.C., Wang S.J. (2017). Exploring resting-state EEG complexity before migraine attacks. Cephalalgia.

[6] Dobell E., Herold S., Buckley J. (2018). Spreadsheet Error Types and Their Prevalence in a Healthcare Context. J. Organ. End User Comput. (JOEUC).

[7] Xiao F., Ding W. (2019). Divergence measure of Pythagorean fuzzy sets and its application in medical diagnosis. Appl. Soft Comput..

[8] Chatterjee K., Pamucar D., Zavadskas E.K. (2018). Evaluating the performance of suppliers based on using the R’AMATEL-MAIRCA method for green supply chain implementation in electronics industry. J. Clean. Prod..

[9] Keshavarz Ghorabaee M., Amiri M., Zavadskas E.K., Antucheviciene J. (2017). Supplier evaluation and selection in fuzzy environments: A review of MADM approaches. Econ. Res.-Ekon. Istraživanja.

[10] Khatwani G., Srivastava P.R. (2018). Impact of Information Technology on Information Search Channel Selection for Consumers. J. Organ. End User Comput. (JOEUC).

[11] Strang K.D., Vajjhala N.R. (2017). Student resistance to a mandatory learning management system in online supply chain courses. J. Organ. End User Comput. (JOEUC).

[12] He Z., Jiang W. (2018). An evidential dynamical model to predict the interference effect of categorization on decision making. Knowl.-Based Syst..

[13] Deng X., Jiang W. (2019). D number theory based game-theoretic framework in adversarial decision making under a fuzzy environment. Int. J. Approx. Reason..

[14] Seiti H., Hafezalkotob A., Martínez L. (2019). R-numbers, a new risk modeling associated with fuzzy numbers and its application to decision making. Inf. Sci..

[15] Liu Z., Pan Q., Dezert J., Han J., He Y. (2018). Classifier Fusion With Contextual Reliability Evaluation. IEEE Trans. Cybern..

[16] Kang B., Zhang P., Gao Z., Chhipi-Shrestha G., Hewage K., Sadiq R. (2019). Environmental assessment under uncertainty using Dempster–Shafer theory and Z-numbers. J. Ambient Intell. Hum. Comput..

[17] Dahooie J.H., Zavadskas E.K., Abolhasani M., Vanaki A., Turskis Z. (2018). A Novel Approach for Evaluation of Projects Using an Interval–Valued Fuzzy Additive Ratio Assessment ARAS Method: A Case Study of Oil and Gas Well Drilling Projects. Symmetry.

[18] Wang X., Song Y. (2018). Uncertainty measure in evidence theory with its applications. Applied Intelligence.

[19] Fu C., Liu W., Chang W. (2018). Data-driven multiple criteria decision making for diagnosis of thyroid cancer. Annals of Operations Research.

[20] Yazidi A., Herrera-Viedma E. (2017). A new methodology for identifying unreliable sensors in data fusion. Knowl.-Based Syst..

[21] Kang B., Chhipi-Shrestha G., Deng Y., Hewage K., Sadiq R. (2018). Stable strategies analysis based on the utility of Z-number in the evolutionary games. Appl. Math. Comput..

[22] Kang B., Deng Y., Hewage K., Sadiq R. (2019). A Method of Measuring Uncertainty for Z-Number. IEEE Trans. Fuzzy Syst..

[23] Deng X., Deng Y. (2019). D-AHP method with different credibility of information. Soft Comput..

[24] Xiao F. (2019). A multiple criteria decision-making method based on D numbers and belief entropy. Int. J. Fuzzy Syst..

[25] Mo H., Deng Y. (2019). An evaluation for sustainable mobility extended by D numbers. Technol. Econ. Dev. Econ..

[26] Xiao F. (2018). A novel multi-criteria decision making method for assessing health-care waste treatment technologies based on D numbers. Eng. Appl. Artif. Intell..

[27] Mo H., Deng Y. (2018). A New MADA Methodology Based on D Numbers. Int. J. Fuzzy Syst..

[28] Seiti H., Hafezalkotob A. (2019). Developing the R-TOPSIS methodology for risk-based preventive maintenance planning: A case study in rolling mill company. Comput. Ind. Eng..

[29] Fan C., Song Y., Fu Q., Lei L., Wang X. (2018). New Operators for Aggregating Intuitionistic Fuzzy Information With Their Application in Decision Making. IEEE Access.

[30] Herrera F., Herrera-Viedma E., Alonso S., Chiclana F. (2009). Computing with words and decision making. Fuzzy Optim. Decis. Mak..

[31] Xiao F. (2018). A Hybrid Fuzzy Soft Sets Decision Making Method in Medical Diagnosis. IEEE Access.

[32] Mardani A., Nilashi M., Zavadskas E.K., Awang S.R., Zare H., Jamal N.M. (2018). Decision Making Methods Based on Fuzzy Aggregation Operators: Three Decades Review from 1986 to 2017. Int. J. Inf. Technol. Decis. Mak..

[33] Ding W., Lin C.T., Prasad M. (2018). Hierarchical co-evolutionary clustering tree-based rough feature game equilibrium selection and its application in neonatal cerebral cortex MRI. Expert Syst. Appl..

[34] Deng X., Jiang W., Wang Z. (2019). Zero-sum polymatrix games with link uncertainty: A Dempster-Shafer theory solution. Appl. Math. Comput..

[35] Cao Z., Lin C.T. (2018). Inherent Fuzzy Entropy for the Improvement of EEG Complexity Evaluation. IEEE Trans. Fuzzy Syst..

[36] Dong Y., Zhang J., Li Z., Hu Y., Deng Y. (2019). Combination of Evidential Sensor Reports with Distance Function and Belief Entropy in Fault Diagnosis. Int. J. Comput. Commun. Control.

[37] Ding W., Lin C.T., Chen S., Zhang X., Hu B. (2018). Multiagent-consensus-MapReduce-based attribute reduction using co-evolutionary quantum PSO for big data applications. Neurocomputing.

[38] Dempster A.P. (2008). Upper and Lower Probabilities Induced by a Multivalued Mapping. Classic Works of the Dempster–Shafer Theory of Belief Functions.

[39] Shafer G. (1976). A Mathematical Theory of Evidence.

[40] Su X., Li L., Qian H., Sankaran M., Deng Y. (2019). A new rule to combine dependent bodies of evidence. Soft Computing.

[41] Gong Y., Su X., Qian H., Yang N. (2018). Research on fault diagnosis methods for the reactor coolant system of nuclear power plant based on D-S evidence theory. Ann. Nucl. Energy.

[42] Yin L., Deng Y. (2018). Measuring transferring similarity via local information. Phys. A Stat. Mech. Appl..

[43] Fei L., Deng Y., Hu Y. (2018). DS-VIKOR: A New Multi-criteria Decision-Making Method for Supplier Selection. Int. J. Fuzzy Syst..

[44] Sun R., Deng Y. (2019). A new method to identify incomplete frame of discernment in evidence theory. IEEE Access.

[45] Jiang W., Huang C., Deng X. (2019). A new probability transformation method based on a correlation coefficient of belief functions. Int. J. Intell. Syst..

[46] Su X., Li L., Shi F., Qian H. (2018). Research on the Fusion of Dependent Evidence Based on Mutual Information. IEEE Access.

[47] Yager R.R. (1987). On the Dempster–Shafer framework and new combination rules. Inf. Sci..

[48] Smets P. (1990). The Combination of Evidence in the Transferable Belief Model. IEEE Trans.

[49] Lefevre E., Colot O., Vannoorenberghe P. (2002). Belief function combination and conflict management. Inf. Fusion.

[50] Sun Q., Ye X., Gu W. (2000). A New Combination Rules of Evidence Theory. Acta Electron. Sin..

[51] Li B., Bo W., Wei J., Huang Y., Guo Z. (2001). Efficient combination rule of evidence theory. Object Detection, Classification, & Tracking Technologies.

[52] Murphy C.K. (2000). Combining belief functions when evidence conflicts. Decis. Support Syst..

[53] Deng Y., Shi W., Zhu Z., Liu Q. (2005). Combining belief functions based on distance of evidence. Decis. Support Syst..

[54] Jiang W., Wei B., Xie C., Zhou D. (2016). An evidential sensor fusion method in fault diagnosis. Adv. Mech. Eng..

[55] Zhang Z., Liu T., Chen D., Zhang W. (2014). Novel Algorithm for Identifying and Fusing Conflicting Data in Wireless Sensor Networks. Sensors.

[56] Yun L., Li Y., Yin X., Zheng D. (2018). Multisensor Fault Diagnosis Modeling Based on the Evidence Theory. IEEE Trans. Reliab..

[57] Song Y., Deng Y. (2019). A new method to measure the divergence in evidential sensor data fusion. Int. J. Distrib. Sens. Netw..

[58] Yin L., Deng X., Deng Y. (2019). The negation of a basic probability assignment. IEEE Trans. Fuzzy Syst..

[59] Deng X., Jiang W. (2018). Dependence assessment in human reliability analysis using an evidential network approach extended by belief rules and uncertainty measures. Ann. Nucl. Energy.

[60] Yager R.R., Elmore P., Petry F. (2017). Soft likelihood functions in combining evidence. Inf. Fusion.

[61] Zhang W., Deng Y. (2018). Combining conflicting evidence using the DEMATEL method. Soft Comput..

[62] Jiang W., Hu W. (2018). An improved soft likelihood function for Dempster-Shafer belief structures. Int. J. Intell. Syst..

[63] Knuth K.H., Habeck M., Malakar N.K., Mubeen A.M., Placek B. (2015). Bayesian evidence and model selection. Digit. Signal Process..

[64] Knuth K., Placek B., Angerhausen D., Carter J., D’Angelo B., Gai A., Carado B. (2017). EXONEST: The Bayesian Exoplanetary Explorer. Entropy.

[65] Yang J., Xu D. (2013). Evidential reasoning rule for evidence combination. Artif. Intell..

[66] Chatterjee K., Zavadskas E.K., Tamosaitiene J., Adhikary K., Kar S. (2018). A Hybrid MCDM Technique for Risk Management in Construction Projects. Symmetry.

[67] Han Y., Deng Y. (2018). A hybrid intelligent model for Assessment of critical success factors in high risk emergency system. J. Ambient Intell. Humaniz. Comput..

[68] Xu H., Deng Y. (2019). Dependent Evidence Combination Based on DEMATEL Method. Int. J. Intell. Syst..

[69] Song Y., Wang X., Zhu J., Lei L. (2018). Sensor dynamic reliability evaluation based on evidence theory and intuitionistic fuzzy sets. Appl. Intell..

[70] Fei L., Deng Y. (2019). A new divergence measure for basic probability assignment and its applications in extremely uncertain environments. Int. J. Intell. Syst..

[71] Han Y., Deng Y. (2019). A novel matrix game with payoffs of Maxitive Belief Structure. Int. J. Intell. Syst..

[72] He Z., Jiang W. (2018). An evidential Markov decision making model. Inf. Sci..

[73] Han Y., Deng Y. (2018). An Evidential Fractal AHP target recognition method. Def. Sci. J..

[74] Li Z., Chen L. (2019). A novel evidential FMEA method by integrating fuzzy belief structure and grey relational projection method. Eng. Appl. Artif. Intell..

[75] Chen L., Yu H. (2019). Emergency Alternative Selection Based on an E-IFWA Approach. IEEE Access.

[76] Chen L., Deng Y. (2018). A new failure mode and effects analysis model using Dempster–Shafer evidence theory and grey relational projection method. Eng. Appl. Artif. Intell..

[77] Chen L., Deng X. (2018). A Modified Method for Evaluating Sustainable Transport Solutions Based on AHP and Dempster–Shafer Evidence Theory. Appl. Sci..

[78] Jiang W. (2018). A correlation coefficient for belief functions. Int. J. Approx. Reason..

[79] Deng X. (2017). Analyzing the monotonicity of belief interval based uncertainty measures in belief function theory. Int. J. Intell. Syst..

[80] Zhu J., Wang X., Song Y. (2018). Evaluating the Reliability Coefficient of a Sensor Based on the Training Data Within the Framework of Evidence Theory. IEEE Access.

[81] Fu C., Chang W., Xue M., Yang S. (2019). Multiple criteria group decision making with belief distributions and distributed preference relations. Eur. J. Oper. Res..

[82] Seiti H., Hafezalkotob A., Naja S., Khalaj M. (2018). A risk-based fuzzy evidential framework for FMEA analysis under uncertainty: An interval-valued DS approach. J. Intell. Fuzzy Syst..

[83] Zhang H., Deng Y. (2018). Engine fault diagnosis based on sensor data fusion considering information quality and evidence theory. Adv. Mech. Eng..

[84] Deng Y. (2016). Deng entropy. Chaos Solitons Fractals.

[85] Shannon C.E. (2001). A Mathematical Theory of Communication. SIGMOBILE Mob. Comput. Commun. Rev..

[86] Li Y., Deng Y. (2018). Generalized Ordered Propositions Fusion Based on Belief Entropy. Int. J. Comput. Commun. Control.

[87] Cui H., Liu Q., Zhang J., Kang B. (2019). An improved Deng entropy and its application in pattern recognition. IEEE Access.

[88] Xiao F. (2019). Multi-sensor data fusion based on the belief divergence measure of evidence and the belief entropy. Inf. Fusion.

[89] Li W., Guo K. (2010). Combination rules of D-S evidence theory and conflict problem. Syst. Eng. Theory Pract..

